# Stress and Metabolomics for Prediction of Spontaneous Preterm Birth: A Prospective Nested Case-Control Study in a Tertiary Hospital

**DOI:** 10.3389/fped.2021.670382

**Published:** 2021-09-07

**Authors:** Dongni Huang, Zheng Liu, Xiyao Liu, Yuxiang Bai, Mengshi Wu, Xin Luo, Hongbo Qi

**Affiliations:** ^1^Department of Obstetrics, The First Affiliated Hospital of Chongqing Medical University, Chongqing, China; ^2^China-Canada-New Zealand Joint Laboratory of Maternal and Fetal Medicine, Chongqing Medical University, Chongqing, China; ^3^Joint International Research Laboratory of Reproduction and Development of Chinese Ministry of Education, Chongqing Medical University, Chongqing, China; ^4^Department of Obstetrics, Chongqing Health Center for Women and Children, Chongqing, China

**Keywords:** spontaneous preterm birth, cortisol, metabolomics, predictive model, serum

## Abstract

Spontaneous preterm birth (sPTB) is the leading cause of infant morbidity and mortality worldwide. Deficiency of effective predict methods is an urgent problem that needs to be solved. Numbers of researchers spare no efforts to investigate differential indicators. To evaluate the value of the differential indicators, a prospective nested case-control study was carried out. Among an overall cohort of 1,050 pregnancies, 20 sPTB pregnancies, and 20 full-term pregnancies were enrolled in this study. Participants were followed-up until labor. The psychological profile was evaluated utilizing the Zung Self-Rating Depression Scale at 11–14 weeks. Stress-related biomarker-cortisol and metabolites were detected by Electrochemiluminescence Immunoassay (ECLIA) and Gas Chromatography-Mass Spectrometry (GC-MS) in serum samples during pregnancy, respectively. The expression level of cortisol was up-regulated in serum and the score of the Zung Self-Rating Depression Scale was significantly higher in the sPTB group when compared to the control group. Note that, 29 metabolomics were differentially expressed between the sPTB group and the control group. The scores of the Zung Self-Rating Depression Scale, the level of cortisol, Eicosane, methyltetradecanoate, and stearic acid in serum were selected to establish the model with lasso logistic regression. Validation of the model yielded an optimum corrected AUC value of 89.5%, 95% CI: 0.8006–0.9889 with a sensitivity of 100.0%, and specificity of 78.9%. In conclusion, this study establishes a prediction model of sPTB with five variables, which may predict sPTB more accurately and sensitively in the second trimester.

## Introduction

Spontaneous preterm birth (sPTB), defined as delivery before 37 weeks of gestation, is a prevalent complication of pregnancy. About 15 million preterm infants born each year, accounting for ~10.6% of all live births worldwide, and the incidence of sPTB in China between 2015 and 2016 was 7.3% (1, 2). Furthermore, those preterm neonates who do survive have higher rates of short-term and long-term morbidities. Comparing with full-term infants, preterm infants may have higher incidences of some common complications, including respiratory distress syndrome, feeding difficulties, hypoxic ischemic encephalopathy, and developmental disabilities (3–5). In addition, threatened preterm delivery increases the possibility of final preterm delivery, and there are no clinical symptoms and signs in the early stage and lacks of accurate and specific predictive indicators in clinical practice. Successful identification of at-risk women at an early stage will be a major leap forward in the discovery science of preterm birth by allowing identification of the most informative population for pathophysiology understanding and intervention development. Therefore, there is an urgent need to identify other potential factors involved in sPTB to establish a new model for prediction.

sPTB has been linked to a complex cluster of overlapping biomedical, social and psychological factors (6). Among them, stress is widely considered as a contributor of preterm birth. Some studies have shown that stress, as indicated by stressful life events, perceived stress, and depression, is higher among women who go on to deliver preterm. Furthermore, stress can be triggered from many different sources, including low socioeconomic status, elderly age, and social support (7). The maturation of hypothalamic–pituitary–adrenal axis (HPA) axis can create a pre-control hormone environment for the myometrium and enhance uterine contraction. In addition, glucocorticoid induces the expression of related enzymes in placenta, which converts progesterone into estrogen. Cortisol, the hormonal endpoint of physiologic stress-related activation of the hypothalamic–pituitary–adrenal axis, has been associated with fetal matureness and parturition by stimulating prostaglandin secretion and promoting uterine contraction, and is often cited as a potential mediator between stress and pregnancy outcomes. Cortisol fluctuates rapidly in response to even subtle changes in the environment and single serum measurement do not reflect chronic levels (8). Additionally, previous studies carried out in PTB women found that high cortisol levels were a reliable predictor for preterm birth at 48 h, but the predictive index is dull and the accuracy is not high (9). Hence, it is necessary to combine multiple factors to improve the accuracy and efficiency of prediction.

Metabolomics, the investigation of low molecular weight biochemical (metabolites) in cells, tissues, or organisms, which developed from genomics, transcriptomics, and proteomics, is a comprehensive analysis of metabolites investigated to search for disease-specific metabolic signals, which could be used as biomarkers (10, 11). Metabolomics has previously been successfully utilized in the diagnosis of pregnancy complications, such as preeclampsia and fetal growth restriction (12). Mass-spectrometry-based metabolic profiling is increasingly used to uncover new biomarkers for diagnosis, prognosis, pathogenesis clarification and potential therapeutic targets for clinical treatment (13, 14). Thus, metabolomics is a powerful tool to find biomarkers. Given this, in this study, we combine stress biomarkers and metabolites to establish a predictive model for sPTB, which might have a crucial effect on preterm birth treatment.

## Materials and Methods

### Sample Collection

**Figure 1:**
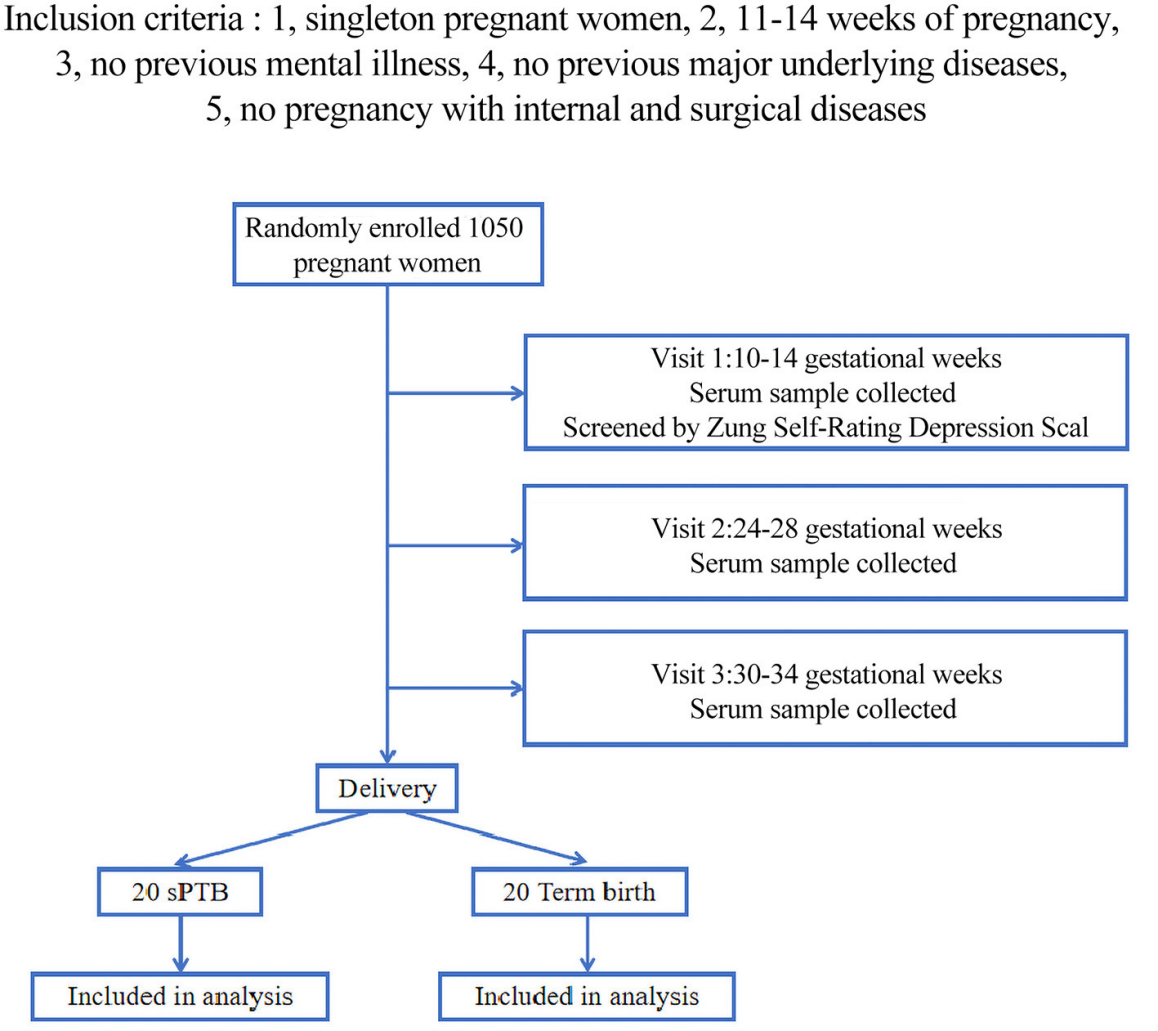
The flow chart of our cohort.

Participants enrolled in the nested case-control study were selected from a prospective cohort. All enrolled pregnancies were followed up from enrollment to 42 days after birth, and their basic and clinical information were collected. This study began recruiting participants in April 2017. If the number of losing follow-up was more than expected, more pregnancies were recruited. The ethics approval was obtained from the Ethics Committee of the First Affiliated Hospital of Chongqing Medical University. All the participants signed informed consent and were enrolled into the study if they were a singleton pregnancy. Then they should be 10–14 weeks of pregnancy, Depressive symptoms of participants were assessed by the Zung Self-Rating Depression Scale (ZSDS) in the first trimester (10–14 gestational weeks). ZSDS ≥ 50 was used as the cut-off score for the presence of depressive symptoms. Bio-samples (serum) were collected at each visiting point. sPTB was defined according to the criteria published by the American College of Obstetrics and Gynecology. All the pregnancies with chronic medical disorders were excluded, including gestation diabetes mellitus, cardiovascular disease, chronic renal disease, collagen disorders, chronic hypertension, and metabolic diseases. Finally, 20 sPTB pregnancies (*n* = 20) and 20 full-term pregnancies (*n* = 20) were enrolled in our study (Figure 1). The samples were obtained from 8:00 a.m. to 10:00 a.m. in the hospital. After obtaining the samples, they were transferred to the laboratory with ice boxes within 1 h and centrifuged at 3,000 rpm for 10 min at 4°C. Serum samples were kept at −80°C until analysis.

### Laboratory Analysis

Serum cortisol levels were detected by a single stage electrochemiluminescence immunoassay (ECLIA, Cobas 8000 Roche Diagnostics Scandinavia AB). The method had a coefficient of variation (CV) of 7% at 100 nmol/L, 5% at 570 nmol/L, and 5% at 990 nmol/L.

### Sample Preparation and Analysis by GC-MS

To determine the practicability of using the maternal serum metabolome in identifying the asymptomatic women who were at risk of sPTB in the second trimester, GC-MS was performed. Serum samples were thawed and analyzed by GC-MS according to the method described by Li et al. (10). A 100 μL aliquot of serum was centrifuged at 15,000 rpm for 10 min at 4°C. Then, the mixture was obtained by spiking an internal standard (2-chlorophenylalanine, 0.3 mg/mL) into serum and then vortexed for 10 s. This mixture was then extracted with 150 μL of methanol-acetonitrile and vortexed for 30 s, followed by cooling for 10 min at −20°C and then centrifuging at 15,000 rpm for 10 min at 4°C. An aliquot of the 150 μL supernatant was placed in a glass sampling vial and dried under vacuum at room temperature. The residue was first reconstituted in 80 μL of methoxyamine (15 mg/mL in pyridine), then vortexed for 30 s, and kept at 37°C for 90 min. Finally, 80 μL of bis(trimethylsilyl) trifluoroacetamide (BSTFA) (1% trimethylchlorosilane) and 20 μL of n-hexane were added, and the extract was kept at 70°C for 60 min. Derivatized extract was separated by a GC7890 chromatography system using a ZB-1701 GC capillary column (30 m × 250 μm id × 0.15 μm with a 5 m guard column, Phenomenex) and analyzed by a MSD5975 mass spectrometry (Agilent, California, USA) with electron impact ionization via electron emission at 70 eV. The GC temperature program and MS parameters were set according to the protocol described in Smart et al. (15).

### Metabolites Validation, Data Mining, and Statistical Analysis

Automated Mass Spectral Deconvolution & Identification System software was employed for metabolite deconvolution. The compounds were identified by comparing the MS fragmentation patterns and respective GC retention time to an in-house MS library established using chemical standards. The remaining putative compounds were identified using a commercial NIST mass spectral library. The MassOmics XCMS R-based script was used to extract the relative concentration of the metabolites through the peak height of the most abundant fragmented ion mass. To improve quantitative robustness and minimize human and instrumental variabilities, the relative abundances of the identified compounds were normalized in the order of multiple internal standards, median centering-batch correction via QC samples. Student's *t*-test, non-parametric Mann-Whitney U test, Chi-square test, and Fisher's exact test were performed in R to compare clinical characteristics between the control and the sPTB group. Before metabolomic statistical analysis, the serum metabolite profiles were adjusted to Gaussian distribution through log transformation and Pareto scaling. Partial least squares discriminant analysis (PLS-DA) was performed to compare serum metabolome profiles between two groups using the Metaboanalyst 3.0 package for R (16).

### Predictive Model Establishment

#### Data Standardization

In the original data, each variable was continuous. However, the data were various and differed in units. Hence, the model parameter estimation coefficients were standardized before analysis in order to overcome the influence of the dimension and make it comparable.

- Lasso logistic regression: the glmnet package of R software (version 3.6.1) was employed to implement Lasso logistic regression, and the value of λ was determined through cross-validation.
- Only Lasso was used for feature selection, and then a supporting vector machine prediction model was established using radial basis function (RBF), polynomial, and Sigmoid kernel function. The independent prediction ability of the model was tested by Leave-One-Out method (LOO): Take one sample at a time from the n samples as the test set, and use the remaining *n*-1 samples to form the training set.

## Results

### Participants

The differences of demographic and clinical characteristics between both groups were presented in Table 1. This information revealed that both groups had similar BMI and age. The difference between the two groups was not statistically significant. Additionally, the association between the lower birth weight infants and the sPTB pregnancies was found. Participants complicated with sPTB had lower birth weight infants and higher ZUNG scores (*P* < 0.001), which indicated that higher ZUNG scores was a potential clinical predictor for sPTB.

**Table 1:** Medians or Counts (%) for demographic and clinical variables.

**Variables**	**Term birth (*n* = 20)**	**Spontaneous preterm** **birth (*n* = 20)**
Maternal age (years)^*^	33.10 ± 1.275	32.50 ± 1.222
Maternal BMI^*^	25.94 ± 0.6721	27.13 ± 0.6119
ZUNG score^#^	42.56 ± 1.123	49.19 ± 0.6494
Birth weight (g)^#^	3381 ± 66.21	2619 ± 116.1
**Birth gender**		
Boys	9 (45%)	8 (40%)
Girls	11 (55%)	12 (60%)
**Gestational age at delivery (weeks)**		
<28	N/A	0 (0%)
28–32		1 (5%)
32–34		3 (15%)
34–37		16 (80%)
**Preterm premature rupture of membranes**		
Yes	N/A	10 (50%)
No		10 (50%)

*BMI, body mass index*.

*Data are presented as mean ± SD*.

* *t-test P-value 0.7359 for age, 0.1977 for BMI, respectively*.

# *t-test P-value < 0.0001 for ZUNG Score and birth weight*.

### Expression of Cortisol in Serum Samples During Pregnancy

**Figure 2:**
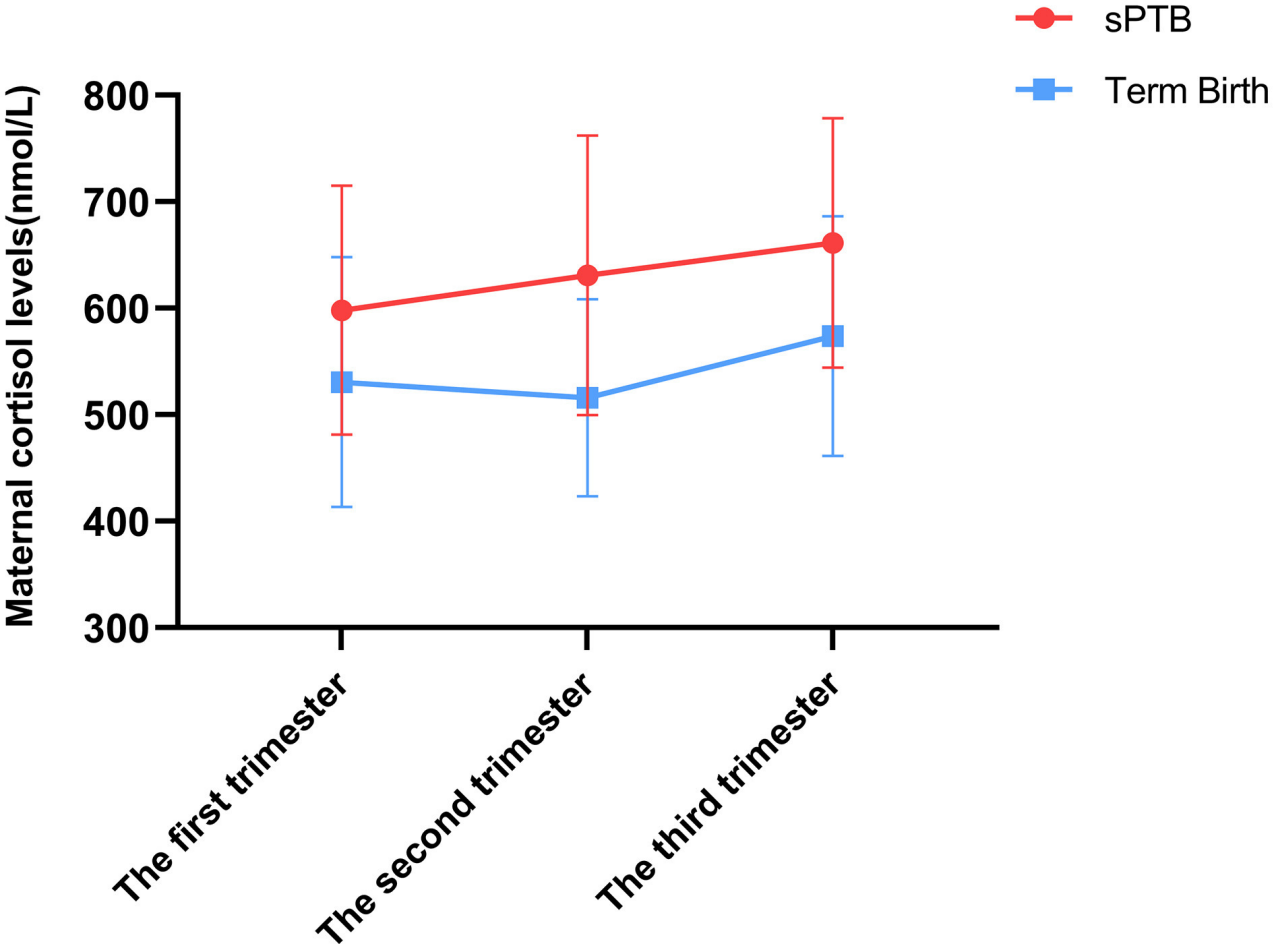
The expression levels of cortisol in maternal serum complicated with sPTB and full-term pregnancies. The cortisol level was measured by ECLIA. Statistical significance between three comparisons was determined utilizing Student's *t*-test. The sPTB group had higher cortisol level in d and third trimester, but no difference in the first trimester.

Firstly, the expression level of cortisol was detected in serum samples. According to our results, the expression level was found to increase from the first trimester to the third trimester. After we analyzed the data, the association between sPTB and maternal cortisol levels across pregnancy was detected. In the first trimester, there was no significant difference in cortisol levels between both groups (Term vs. sPTB, *P* = 0.081). However, in the second trimester, cortisol levels of serum in the sPTB group were detected dramatically higher than those in the full-term group (Term vs. sPTB, *P* = 0.005). Moreover, such differences still persisted in the third trimester (Term vs. sPTB, *P* = 0.043) (Figure 2).

### Metabolites Significantly Associated With sPTB

**Figure 3:**
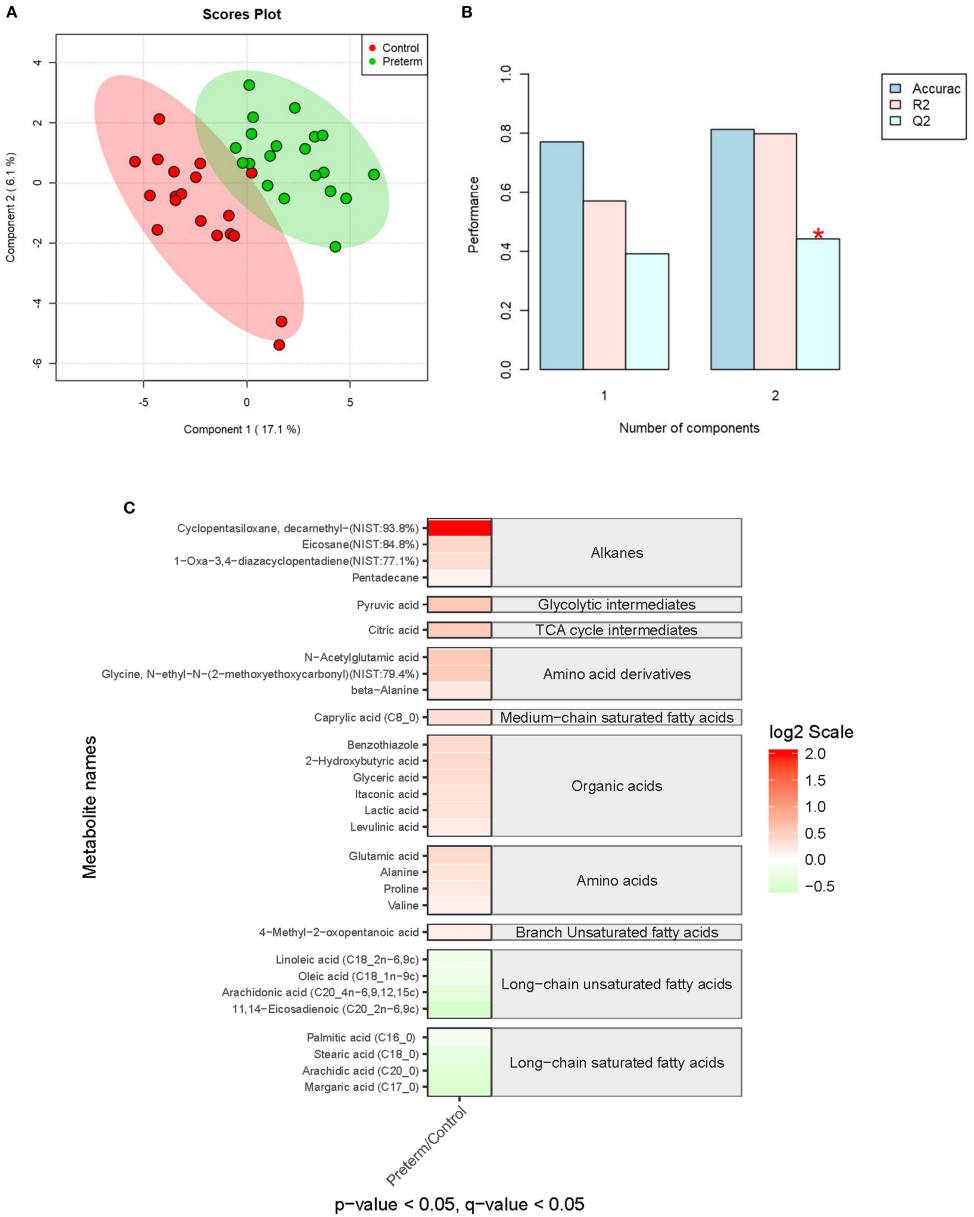
Partial least squares discriminant analysis (PLS-DA) and the heatmap of the serum metabolome between the sPTB group and the full-term group. **(A,B)** PLS-DA of the serum metabolome between the sPTB group and the full-term group, including a measure of prediction model performance. **(C)** The heatmap shows the differentially expressed metabolites. The relative concentrations of serum metabolites were demonstrated through log_2_ scale. The red color blocks indicate a higher expression level, and the green color blocks indicate a lower expression level. Only the metabolites with a *p*-value of <0.05 and a *q*-value of <0.05 were displayed.

A total of 119 compounds were identified from the in-house MCF spectral library and NIST library (https://www.nist.gov/nist-research-library). The partial least squares discriminant analysis (PLS-DA) showed the most distinct separations between the sPTB group and the full-term group (Figures 3A,B). As shown in Figure 3C, 29 serum metabolites were detected significantly different in concentrations between the two groups with *p*-value and *q*-value < 0.05 and 0.05, respectively. Among them, 18 metabolites were validated significantly upregulated in the sPTB group includes 4 alkanes, 7 amino acid derivatives, 1 glycolytic intermediate, and 6 prganic acids. Conversely, 11 metabolites were found to be significantly downregulated in the sPTB group includes 4 long-chain saturated fatty acids, 1 Medium-chain saturated fatty acid, 1 TCA cycle intermediate, 4 long-chain unsaturated fatty acids, and 1 branch Unsaturated fatty acid.

### Construction and Validation of the Prediction Model for sPTB

**Figure 4:**
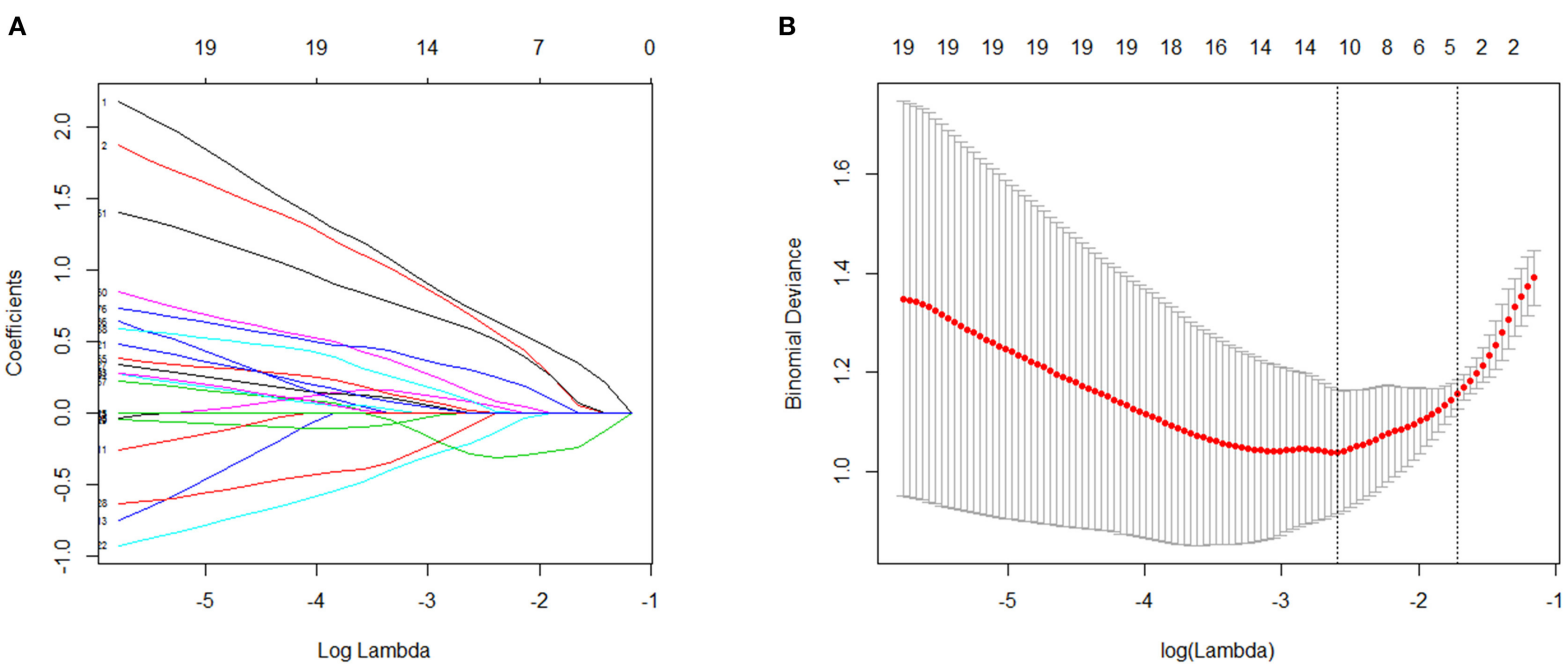
Validation screened by Lasso. **(A)** The changes of coefficients with parameters were plotted in Lasso regression. **(B)** The change diagram of binomial device with parameter ln λ was drawn through 3-folds cross validation.

To further shortlist the significant metabolites that might have clinical implications, Lasso was carried out for all metabolites. The results showed that when λ _min_ = 0.07439764, that was, log (λ _min_) = −2.598331 (left dotted line in Figure 4A), the binomial device reached the minimum value. In addition, λ _1se_ = 0.1800518, that was, log (λ _1se_) = −1.714511 (dotted line on the right side in Figure 4B) was the best λ value corresponding to the simplest model within a standard error of λ _min_. Finally, the coefficient of the selected model was λ _1se_, corresponding to five variables selected to enter the regression model, namely ZUNG score, serum cortisol level, Eicosane level, i-Propyl 12-methyltetradecanoate level, and Stearic acid level. If variable coefficient was a negative number, indicating the negative correlation between the function and the variable, that means the probability of premature delivery risk decreased with the increase of the index value; otherwise, the greater the index value, the higher premature delivery risks (Table 2). Validation of the model yielded an optimum corrected AUC value of 89.5%, 95% CI (0.8006–0.9889) with a sensitivity of 100.0% and specificity of 78.9%. The model's independent prediction ability was tested by LOO after establishing the model using RBF, polynomial, and Sigmoid kernel function with the accuracy of 94.9, 89.7, and 97.4%, respectively, as illustrated in Table 3.

**Table 2:** Standardized regression coefficient of variables.

**Variables**	**Standardized regression coefficient**
Intercept	0.04908751
ZUNG score	0.37593216
Cortisol level (intercept)	0.10916823
i-Propyl 12-methyltetradecanoate level	0.02658327
Eicosane level	0.11962625
Stearic acid level	−0.24982455

**Table 3:** Test the model's independent prediction ability by LOO.

**Function**	**Correct number**	**Accuracy**
RBF	37	94.90%
Poly	35	89.70%
Sigmoid	38	97.40%

## Discussion

To the best of our knowledge, this is the first prospective nested case-control study to investigate whether psychosocial factors and metabolites can predict spontaneous preterm birth in the second trimester. In this study, we found that concentrations of serum cortisol and scores of ZUNG depression scale were positively associated with the risk of sPTB. Moreover, 29 aliphatic acids, expressed differentially between the sPTB and the full-term maternal serum were identified through GC-MS in this study. The strengths of this study lie in the prospective design and the rapid determination of sPTB risk in the second trimester. Besides, validation of the model yields high sensitivity and specificity.

Preterm birth, as a multifactor syndrome, can be triggered by stress (17). Women with likely diagnosis of a major depressive episode are at a 4-fold increased risk of preterm birth (18). Previous studies have found that cortisol levels appeared higher in sPTB compared to term birth. Hence, the high levels of stress biomarker-cortisol may play an important role in the prediction of preterm birth. Additionally, the twin pregnancies who experienced social stress also undergone preterm birth (19). Cortisol, a biomarker for indication of delivery, are capable of being refined by models that incorporate in other biomarkers. However, few predictive models can predict preterm birth in mid-pregnancy by assessing maternal stress and metabolites. Grasso Lucia et al. found that serum cortisol was more sensitive and specific than urine cortisol throughout pregnancy (20). Therefore, serum cortisol was included in this model to ensure the accuracy and reliability of the preliminary model. To explore the relationship between pregnancy complications and prenatal exposure factors, a large pregnancy cohort was established. Therefore, the serum cortisol and metabolic concentrations observed in our study can be considered representative of the general maternal level at some scale. In addition, this prospective study can provide first-hand information of stress and spontaneous preterm birth directly with little bias.

Few studies have applied metabolomic techniques to understand sPTB in asymptomatic pregnant women. A total of 29 aliphatic acids were differentially expressed between the sPTB and the full-term maternal serum in the second trimester by GC-MS. Among them, we further enlisted i-Propyl 12-methyltetradecanoate, Eicosane, and Stearic acid into our model for sPTB predicting to improve its performance over a model with cortisol alone (21). Long chain fatty acids are needed for the development of brain, retina, and nervous system of fetal during the third trimester of pregnancy. Stearic acid (a long chain saturated fatty acid) level was found lower in the preterm birth infants than in the term birth infants (22). Actually, there are very few studies indicating the direct relationship between i-Propyl 12-methyltetradecanoate, Eicosane and preterm birth. Fortunately, Eicosane can be found in tobacco (23), previous studies also have found that Mother quit smoking can effectively reduce the risk of recurrent preterm birth (24). In addition, oxidative stress may lead to degradation of cell membranes by lipid peroxidation, following by conversion of polyunsaturated fatty acids to volatile alkanes. Moreover, the relationship of oxidative stress-associated process and sPTB have also been previously reported. These may indicate why an abnormal increase in Eicosane may predict preterm birth. However, investigation of the potential relationship of i-Propyl 12-methyltetradecanoate and preterm birth is further required.

Though a variety of predictive models of preterm birth have been developed, the inability to meet both sensitivity and specificity in mid-pregnancy is a common problem in current preterm prediction models. For instance, a prediction model of spontaneous delivery within 7 days was developed through the diagnosis of amniotic cavity and/or intra-amniotic inflammation. The models presented a high diagnostic performance, showing an area under curve (AUROC) of 0.86 [95% confidence interval (CI) 0.77–0.95] with a detection rate of spontaneous delivery within 7 days of 87%, a false-positive rate of 33%, negative predictive value of 80%, and negative Likelihood ratio of 0.1908 (25). However, it is an invasive diagnosis, not widely available for screening, and can only predict preterm births up to seven days. In order to improve the prediction efficiency, some researchers changed the modeling method, QUiPP App v.2 (26), while some researchers added the prediction variables, placenta alpha microglobulin-1, phosphorylated insulin-like growth factor binding protein-1, and cervical length, respectively (27). However, serum samples were used from the second trimester to build the model in our study, which could predict preterm birth in earlier time. Besides, five variables were selected to enter the regression model to improve the prediction efficiency.

The limitation of this study is that the sample size we use is small. Furthermore, the prediction model was established by machine learning to expand samples to validate the efficiency of prediction. However, our nested case-control study is still ongoing, we will expand the sample size to further verify the prediction efficiency of the model. Moreover, we plan to verify the different expression level of cortisol in urine and saliva samples among the sPTB and the full-term group, which may provide an idea to further establish a non-invasive prediction model in the future.

## Conclusion

In summary, validation of the prediction model suggests that high scores of Zung Self-Rating Depression Scale, high level of cortisol, Eicosane, methyltetradecanoate and lower level of stearic acid in maternal serum can reliably predict the risk of sPTB in asymptomatic pregnant women in the second trimester.

## Data Availability Statement

The raw data supporting the conclusions of this article will be made available by the authors, without undue reservation.

## Ethics Statement

The studies involving human participants were reviewed and approved by The First Affiliated Hospital of Chongqing Medical University Ethics Committee. The patients/participants provided their written informed consent to participate in this study.

## Author Contributions

DH: conceptualization. DH and ZL: data curation and formal analysis. YB: resources. XLi and MW: software. XLu and HQ: supervision. All authors made substantial contributions to the paper and read and approved the final manuscript.

## Funding

This work was supported by grants from the National Key R&D Program of China (No.2016YFC1000407); the National Natural Science Foundation of China (No.81771614 and No.81771613); the Science and Technology Department of Sichuan Province (No.2020YFQ0006).

## Conflict of Interest

The authors declare that the research was conducted in the absence of any commercial or financial relationships that could be construed as a potential conflict of interest. The reviewer LW declared a shared affiliation, with no collaboration, with the authors to the handling editor at the time of the review.

## Publisher's Note

All claims expressed in this article are solely those of the authors and do not necessarily represent those of their affiliated organizations, or those of the publisher, the editors and the reviewers. Any product that may be evaluated in this article, or claim that may be made by its manufacturer, is not guaranteed or endorsed by the publisher.
